# The Psychometric Properties of the Intergenerational Trauma Socialization Inventory: Development and Validation of a Multidimensional Measure of Intergenerational Transmission of Trauma Among Lao American Families

**DOI:** 10.1007/s42844-026-00236-0

**Published:** 2026-07-29

**Authors:** Miwa Yasui, Eunseok Jeong, Shira Aronson

**Affiliations:** Crown Family School of Social Work, Policy, and Practice, University of Chicago, Chicago, IL USA

**Keywords:** Transmission of trauma, Southeast Asian, Refugees, Mental health, Socialization, Family process

## Abstract

Refugees face significant mental health struggles due to violence, torture, and trauma from war, genocide, and the process of migration. The effects of severe trauma extend beyond direct victims of traumatic experiences, shaping the mental health of future generations. Yet, quantitative measures assessing mechanisms of how parental trauma is transmitted from parents to offspring are limited. This study examines the validity of the Intergenerational Trauma Socialization Inventory (ITSI), a new measure that assesses socialization pathways of trauma transmission, among Lao American young adults. The ITSI demonstrated an adequate five-factor structure in exploratory factor analyses in Study 1with a sample of 89 Lao American young adults. In Study 2, confirmatory factor analyses were conducted with a sample of 223 Lao American young adults to confirm the five-factor structure derived from the EFA. The correlated five factor model revealed the best fit, χ2 (528) = 1038.67, *p*<.05; RMSEA=0.07; CFI=0.90, revealing multiple pathways of trauma transmission: parental silence, observing parental response to past trauma, sharing as instruction, sharing through cultural channels, and internalization of parental trauma. Bivariate correlations indicated that these pathways of trauma transmission were significantly associated with measures of somatic symptoms and depressive symptoms, suggesting concurrent validity. The ITSI also indicated strong reliability (α > 0.84) for all factors. The ITSI can be a helpful measure in assessing pathways of trauma transmission among refugee families and can be used to inform family interventions and community mental health programs to promote health communication pathways in sharing of familial and cultural histories, thereby potentially reducing the impact of trauma on subsequent generations within families and refugee communities.

Intergenerational trauma refers to trauma experienced in one generation that is transmitted to subsequent generations, resulting in various psychological and physical consequences (Sangalang & Vang, 2016). Trauma and its effects are passed down from generation to generation, often in the form of a family legacy, affecting offspring who themselves are not direct victims of trauma (Danieli, 2007). This intergenerational impact may be established through the physical consequences of the trauma, such as epigenetic changes (Yehuda & Lehrner, 2018), as well as through emotional and social processes (Flanagan et al., 2020; Han, 2006). In addition, sociopolitical, cultural, and historical influences also shape the ways in which familial transmission of trauma occurs and affects subsequent generations (Field et al., 2013).

Trauma exposure and its subsequent intergenerational impact can affect the physical and psychological well-being of the children, including vulnerability to stress and trauma, increased engagement in behavioral risk taking, social dysfunction, and impairment in interpersonal relationships (Giladi & Bell, 2013; Yehuda et al., 1998). Southeast Asian (SEA) refugees such as Lao, Cambodian, and Vietnamese refugees have collectively endured compounding trauma and losses due to genocide, war, and political violence, which is woven into the fabric of parental and cultural trauma (Lin et al., 2009; Patel & Nagata, 2021). Among SEA refugees, the nature of the historical trauma varies (e.g., autogenocide for Cambodian Americans, civil war driven by the Cold War for Vietnamese and Lao Americans) and the intergenerational effects of these specific historical traumas are not well understood. To date, only a handful of studies have examined the impact of intergenerational transmission of trauma among SEA refugees. For example, Vaage et al. (2011) reported a link between Vietnamese refugee fathers’ post-traumatic stress disorder (PTSD) at the time of arrival in the host country and the mental health outcomes of the offspring decades later. Field et al. (2011) found parental trauma effects on child depression among Cambodian refugees, which were mediated through family processes. Lao Americans have also endured intergenerational trauma as a result of being caught in the crossfire between the American and Vietnamese military forces that resulted in more than 25% of the population being displaced, and over 360,000 Lao people fleeing the country between 1975 and 1992 (Stuart-Fox, 1997). In the course of migration, Lao Americans faced significant violence and traumatic experiences, including war, systematic oppression, poverty, and stigmatization prior to the resettlement, all of which posed challenges to their mental health, seen in elevated rates of PTSD (19.7%) and elevated rates of major depression diagnoses and suicide ideation (Yang et al., 2018). The intergenerational effects of this trauma are documented, for example, Yang and Dinh (2018) found that 36% of Lao American youth reported lacking positive coping mechanisms for stress and anger and that, consequently, 49% used alcohol as a coping tool. Moreover, 50% of Lao American male focus group participants reported not having a positive adult ally or mentor at home, with participants attributing this lack of support to having parents and elders with unresolved war traumas.

The apparent mental health impact of parental trauma on offspring underscores the importance of understanding the very mechanisms that underlie the intergenerational transmission of trauma. Among various pathways of parental trauma transmission, intra-family trauma communication, which refers to how parents communicate their traumatic experiences to children and make sense of their current mental distress, is a central avenue through which children learn of parental trauma. Guided by societal and cultural norms of communication, families share past trauma with children in a variety of ways, that range from explicit verbal messages to subtle behavioral or verbal cues and gestures (De Haene et al., 2012). Thus, identifying what messages of trauma parents share and how offspring receive messages is critical. However, to our knowledge, quantitative measures of this socialization process of parental trauma are lacking. This current study seeks to identify specific mechanisms of parent transmission of trauma within SEA refugee families through the development and testing of a new measure of parental socialization of trauma.

## Intergenerational Trauma: Transmission through Intra-Familial Communication

According to theories of trauma transmission, children learn about parental trauma through various modes of parental communication, including explicit or open communication that includes unregulated, retelling of parental trauma histories (i.e., unfiltered speech) to modulated disclosure that may involve parental instruction regarding survival (e.g. Danieli, 1985; De Haene et al., 2010) and parental silence (De Haene et al., 2012; Mor, 1990; Rousseau & Drapeau, 1998). In response to hearing parental experiences of trauma, trauma transmission theories purport that children integrate these parental messages of trauma to connect with and maintain ties with their parents (Mor, 1990).

### Socialization of Trauma Through Parental Silence

Parental silence of trauma, also referred to as the conspiracy of silence, is when parents never share their experiences from the past or the effects of their trauma with their children (Danieli, 1998; Dalgaard et al., 2016; Mor, 1990). As a parental socialization process, parental silence involves the mechanisms of (a) what and how parents refrain from sharing about their past trauma, and (b) how the offspring experience and understand their parents’ silence (Bar-on, 1996). Parents engage in silence for a variety of reasons, such as protecting their children from negative effects of their trauma (Angel et al., 2001; Dalgaard et al., 2016), ensuring that their trauma does not cause their children to feel “othered” (Nagata & Cheng, 2003), having a fear of burdening others (Weine et al., 2004), experiencing feelings of guilt or shame about their trauma (Braga et al., 2012), or protecting themselves from re-experiencing the trauma (Lin et al., 2009). In turn, offspring interpret parental silence as an implicit message not to ask questions about parental trauma histories, despite awareness of its effects on parental mental health and the offspring’s desire to understand their parents’ histories (Dalgaard et al., 2016). Even when parents are silent about their trauma histories, children may still witness their parents’ trauma responses (e.g., PTSD symptoms), which can result in internalization of trauma responses (Dalgaard et al., 2016).

Parental silence is theorized to contribute to the intergenerational transmission of trauma, impacting offspring outcomes in varied ways. Offspring of Holocaust survivors noted that when parents are silent about their past, trauma experiences become family secrets that are acknowledged yet never discussed across generations (Lev-Wiesel & Amir, 2006). This absence of communication about parental trauma is related to greater levels of interpersonal distress (Lichtman, 1984). In contrast, Angel et al. (2001) found that 70% of Bosnian refugee parents who engaged in silence about the war reported that their children were better adjusted compared with children of parents who shared their past trauma. The authors suggested that in cultural contexts where disclosure of emotional or traumatic experiences is discouraged, parental sharing was not beneficial, contrary to existing findings. Nagata and Cheng (2003) found that only 44.5% of Japanese American families, compared with 94% of African American families, had open communication with their children about race-related trauma, indicating that culturally specific familial and communication processes may guide trauma transmission.

### Instruction and Culture as Channels in Open Family Communication on Trauma

As noted above, trauma transmission theories describe varied pathways of open communication about parental trauma, including as an avenue for (a) parents’ expression and response to trauma and (b) intentional communication of past trauma by parents for the purpose of instructing or instilling culture, values, and hope in the next generation (Danieli, 1998; Mor, 1990). Supporting the latter pathway, among families of survivors of the Armenian genocide, messages of parental trauma included retelling stories of survival, horror, death, and anger with the purpose of allowing survivors and the culture as a whole to reconnect with lost love objects (Esmaeili, 2011). Bosnian refugee parents reported sharing painful trauma narratives and happy past memories to build trust, express emotions, and instill hope for rebuilding their future (Weine et al., 2004). Nagata and Cheng (2003) found that for former Japanese internees, although communication about trauma was in general, rare, when parents engaged in explicit sharing, the messages emphasized taking pride in one’s culture or preparing children for the realities of discrimination. While not explicitly described within trauma transmission theories, these studies suggest the centrality of instilling cultural pride and connectedness - which is reflective of cultural socialization, a mode of ethnic-racial socialization (Hughes et al., 2006) - as one of the primary intentions for why parents communicate about historical and parental past trauma. Rousseau and Drapeau (1998) have noted that culture is a channel through which the impact of trauma is passed down from parents to their offspring, often through forms of familial discourse on trauma.

The psychological effects of open communication of parental trauma on children’s mental health are mixed. Among families of Holocaust survivors, Lichtman (1984) found that maternal openness and higher frequency of communication about trauma were linked to negative personality traits in the second generation. Similarly, studies have found open communication of trauma to be associated with increases in internalizing problems in children (Angel et al., 2001; Montgomery, 1998). However, other studies illustrate the benefits of open communication; for example, among children of refugee torture survivors, children whose parents were less open in sharing about their experiences of imprisonment, torture, and escape had poorer emotional adjustment compared with those whose parents openly shared (Montgomery et al., 1992). Moreover, open communication that is modulated and tailored to the child’s developmental stage and understanding, such as using humor to cushion the impact of the trauma message, is found to be protective (Braga et al., 2012; Dalgaard & Montgomery, 2015). For example, Middle Eastern refugee children who received modulated disclosure of parental trauma were more likely to have a secure attachment compared with those whose parents were silent about their trauma (Dalgaard et al., 2016).

Reflective of the unfiltered or unregulated mode of open communication, parental trauma may also be transmitted implicitly, sometimes outside the parent’s awareness but *observed* and recognized by the child (Dalgaard et al., 2016). These may include overhearing parental conversations about past trauma or children receiving snippets of information about their parents’ trauma through unintended messages, such as when parents are tired and accidentally make veiled references or have outbursts of grief (Braga et al., 2012; Okner & Flaherty, 1989). Lin et al. (2009) noted that in Cambodian American families, children learned about trauma by overhearing their parents’ conversations. This type of communication can be confusing and ambivalent for children, who are often incapable of understanding their parents’ behavior (Dekel & Goldblatt, 2008).

### Internalization of Parental Trauma

While existing literature has predominantly examined socialization pathways of trauma from parent to child, intergenerational transmission of trauma is also dependent on how offspring receive and integrate parental messages of trauma. Theories on trauma transmission indicate that as children learn of their parents’ trauma experiences, they simultaneously attempt to grasp parental experiences as a means of connecting with and identifying with the parent (Mor, 1990). For example, the child may attempt to explain parental experiences of survival (e.g., Braga et al., 2012); help parents cope and manage the effects of trauma (Shafet, 1994); or to manage the experience of guilt, victimization, (Braga et al., 2012), mistrust or fear of others (Bezo & Maggi, 2015). The internalization of parental trauma has been found to impact offspring’s mental health, including depression and anxiety (Fossion et al., 2015; Shafet, 1994).

## Present Study: Socialization Process of Parental Trauma

**Fig. 1:**
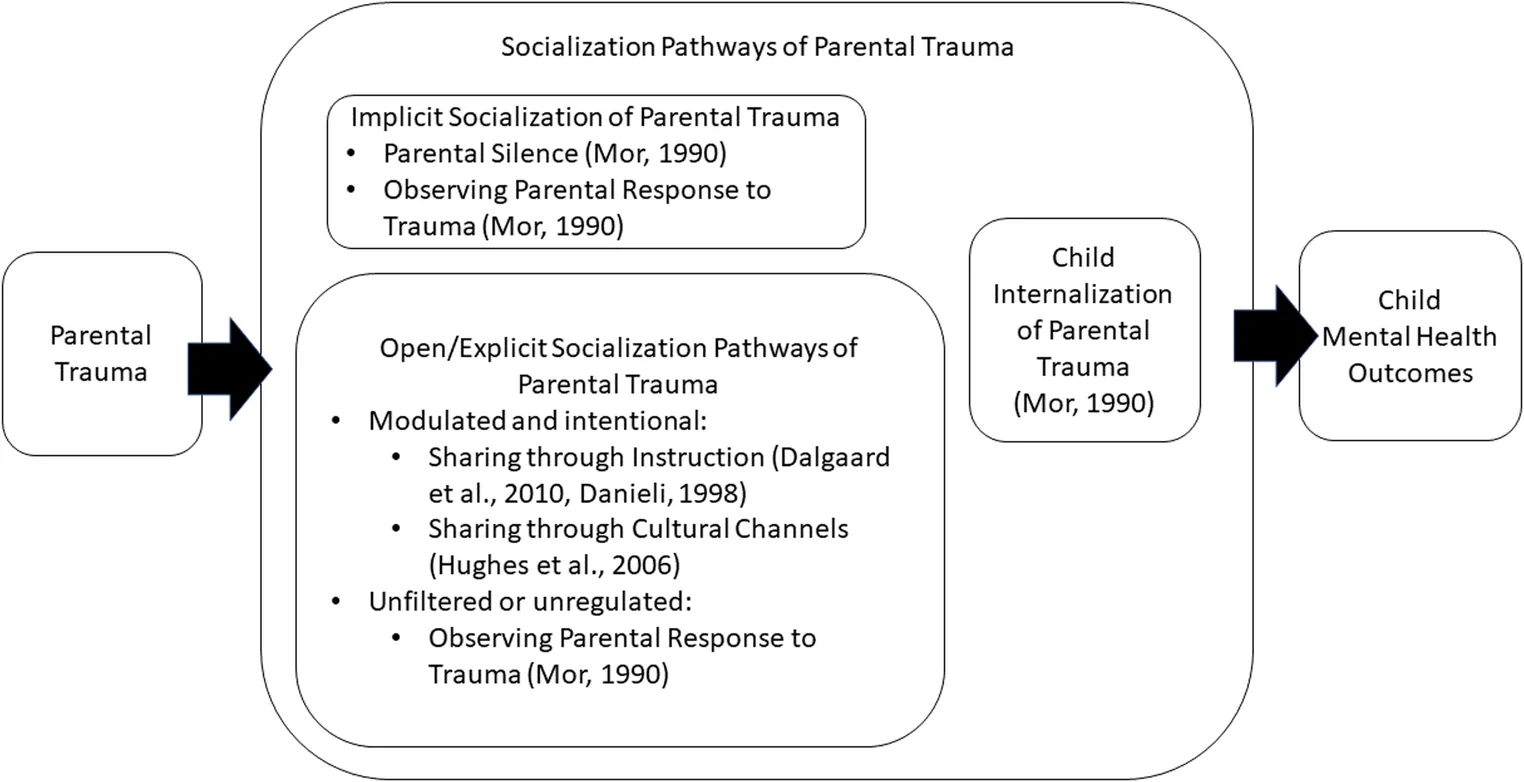
Parental socialization of trauma and informing theories

 Existing frameworks have examined the transmission of trauma through intrafamilial communication (Dalgaard et al., 2016), attachment styles (Sagi-Schwartz et al., 2003), parenting practices (Field et al., 2013), and psychobiological processes and genetics (Yehuda & Lehrner, 2018). Parental socialization, a central socializing mechanism that shapes how children develop values, beliefs, and practices, may also be a helpful framework for understanding the specific mechanisms by which parental trauma is transmitted to offspring. The framework of parental socialization has served as a basis for understanding the mechanisms of socializing children in a variety of areas, including emotion (e.g., Eisenberg et al., 1998), race and ethnicity (e.g., Hughes et al., 2006), health (e.g., Lees & Tinsley, 2000) and mental health (e.g., Yasui et al., 2022). Parental socialization pathways may also be an avenue through which parental trauma is transmitted from parent to child. Drawing from existing theories on trauma transmission, we conceptualize parental socialization as a primary avenue through which parental trauma may be passed down to children, advancing frameworks of intergenerational trauma transmission (see Fig. 1).

The current study will be one of the first to address gaps in existing literature on the intergenerational transmission of trauma among Southeast Asian refugee families. To date, studies examining the transmission of trauma are largely qualitative in nature. To our knowledge, no quantitative measures currently exist that capture the parental socialization process of trauma transmission. This points to a methodological void in validated measures that assess the transmission of trauma in the current literature.

Therefore, the present study sought to develop the Intergenerational Trauma Socialization Inventory (ITSI), a new measure of intergenerational transmission of trauma, that assesses parental socialization pathways through which parental trauma is transmitted to offspring among Lao Americans. The ITSI was developed guided by an extensive review of the literature and qualitative data from focus groups with Lao, Cambodian, and Vietnamese American young adults for another study that examined mental health beliefs and help-seeking (Yasui et al. 2022, 2023a, b). The aim of the current study is to (1) develop and validate the ITSI to assess parental socialization processes of trauma transmission, (2) evaluate the factor structures of the ITSI, (3) confirm the factor structures derived and examine the measure’s psychometric properties, and (4) examine interrelations among transmission of trauma and mental health outcomes among Lao American adult offspring of trauma survivors. In Study 1, we describe the development of the new measure, including item generation, reliability analysis, scale refinement, and initial explanatory factor analysis (EFA). For Study 2, we tested the fit of the factor structure derived in Study 1 and examined correlations between scales of transmission of trauma and existing measures of mental health.

## Study 1

The goals for Study 1 were to develop and test a multidimensional measure of parental transmission of trauma for Asian American immigrant and refugee populations. Items for this inventory were developed using a multiple-method approach that included an extensive literature review on trauma transmission, qualitative data from focus groups with Asian American community members, and expert review and refinement by experts in Asian American mental health and stakeholders from Asian American communities.

## Methods

### Participants

Data for Study 1 were drawn from 88 Lao American young adults, who were a subset of a sample originally collected for a study examining the intergenerational transmission of trauma among Asian American families. Participants were 42% male, with roughly 15% reporting that their household income was less than $35,000 per year, and more than 84% had higher than a high school education. Approximately 79.5% are American-born, and the average age of the participants was 31 (SD = 6.49), with an age range of 18 to 43 (see Table 1).

**Table 1:** Descriptives of Study 1 Sample

	Sample size (*n* = 88)
Sex, *n* (%)	
Male	37 (42%)
Female	51 (58%)
Age in years (SD)[Range]	31.05 (6.49) [18–43]
Household Income, n (%)	
Less than 35,000	13 (14.8%)
More than 35,000	75 (85.2%)
Nativity, n (%)	
U.S. born	70 (79.5%)
Foreign born	18 (20.5%)
Education, n (%)	
Less or equal to high school (GED)	15 (15.9%)
Higher than high school	73 (84.1%)

### Procedures

For the original study, participants were recruited through a partnership with ethnic community-based organizations, including distributing flyers and making verbal announcements at partnering agencies and organizations. Consenting participants took a 60-minute survey in English or Lao, based on their preference and in either online or paper format. All participants were compensated $25 for their time. Study procedures were approved by the University of Chicago Institutional Review Board (IRB protocol # IRB18-0889).

### Measures

#### New Measure of Intergenerational Transmission of Trauma for Immigrants and Refugees

The new measure of the intergenerational transmission of trauma for immigrants and refugees was developed as part of an ongoing original research study that examines cultural and contextual influences on mental health and help-seeking among Asian American (i.e., Lao, Cambodian, Vietnamese, Chinese) immigrant youth and families. The new inventory was developed using a multiple-method approach that involved focus groups, extensive literature review, and expert panel review. For this study, qualitative data from 8 focus groups with youth and adult self-identifying as South-East Asian American was used. Focus groups consisted of between 5 and 12 people, eligibility criteria included the following: (a) self-identifies as East or South-East Asian, (b) between the ages of 12 to 17 for youth and above 18 years old for adults, (c) are 1.5 or 2nd generation. An inductive approach was used, which included open coding and line by line coding of the focus group transcripts to identify emerging codes and themes. These were reviewed, refined, and consolidated into a codebook by a team of 8 coders. All coders self-identified as Asian or Asian American, and included undergraduate students in psychology, masters level students in social sciences and social work, doctoral students in social work, research staff, and the principal investigator of the study. All transcripts were double-coded by two independent coders and any discrepancies in coding were reviewed by the entire coding team in weekly consensus meetings and resolved by reviewing definitions of the codes and discussing the differences in codes. Interrater reliability (kappa) was calculated for each code and then averaged, resulting in (κ) = 0.87, indicating consistency across coders.

Guided by the extensive literature review, codes from the codebook that pertained to themes of parental trauma and intergenerational transmission were selected for the development of preliminary items. Specifically, codes that aligned with transmission pathways identified from existing frameworks of intergenerational trauma, such as the conspiracy of silence (Daniele, 1988), parental silence and open disclosure (DeHaene et al., 2010), and implicit communication (Dalgaard et al., 2016), were selected. In addition, the focus group data revealed a pathway of socialization through culture, a pathway currently not included in frameworks of intergenerational trauma. To guide the selection of appropriate codes through cultural pathways, the literature on ethnic-racial socialization was reviewed, and codes that aligned with cultural socialization processes were selected for item development. After selecting relevant codes, participant words from the focus group transcripts related to the selected codes were reviewed and integrated into the wording for item construction. For example, the item “When I asked my parents about their past, they tell me that our life here in America is what matters most” was derived from a code on parental dismissal/minimization of past trauma and revised using participant language from transcripts. The developed items were then reviewed and revised by stakeholders in partnering Asian American communities and experts in Asian American mental health. All items used a 5-point Likert scale: (1) “Not at all,” (2) “Not much,” (3) “Moderately,” (4) “Much,” and (5) “Very much.” Since the sample consisted of young adult offspring who were either American-born or migrated before the age of 10, the new measure was administered in English. We present the Intergenerational Trauma Socialization Inventory (ITSI) and its corresponding items in Table 2, along with descriptions of the subscales below.

**Table 2:** Study 1 Exploratory Factor Analysis

Items	Observing Parental Response to Past Trauma	Parental Silence on Past Trauma	Internalization of Parental Trauma	Sharing as Instruction	Sharing Through Cultural Channels
I have secretly seen my parents cry when they are alone	**0.54**	− 0.07	− 0.03	0.04	− 0.05
I have seen families in my community pass down the history of the trauma or hardships they endure to their children and community	**0.76**	− 0.09	− 0.09	0.02	− 0.06
I have seen my family suffer from the memories of trauma, genocide, or migration journey to America	**0.90**	− 0.11	− 0.09	− 0.07	− 0.07
I have seen my parents get upset when they talk about their past	**0.78**	0.09	− 0.05	− 0.06	0.16
I have seen my parents have nightmares about their past	**0.52**	0.17	0.01	0.04	0.27
I have seen my parents struggle silently with the problems of their past	**0.77**	0.15	0.13	0.15	− 0.06
In my family, trauma is unspoken but recognized by everyone	**0.41**	0.31	− 0.22	− 0.09	0.03
My parents are superstitious about things such as nightmares, stories, or events related to people from their past	**0.55**	0.15	− 0.07	0.17	0.00
I feel that it is taboo it is wrong to ask about my parents’ past experiences in their home country or migration journey to America	− 0.06	**0.63**	− 0.19	− 0.09	0.07
My parents do not talk about their experiences before coming to American (e.g., genocide, refugee camps, migration journey)	− 0.03	**0.58**	0.04	0.02	− 0.24
My parents get awkward, tense up, get upset when I ask them about their past experiences in their home country or migration journey to America	0.24	**0.66**	− 0.04	0.01	0.08
My parents have never spoken about the past to anyone	− 0.07	**0.70**	0.00	− 0.01	− 0.25
No one in my family talks about their past experiences before coming to the US (in home country, in migrating to America)	0.12	**0.54**	0.21	− 0.06	− 0.13
When I asked my parents about their past, they tell me that our life here in America is what matters most	0.12	**0.47**	0.14	0.20	0.22
When I asked my parents about their past, they tell me that they have forgotten, don’t remember about the past	0.03	**0.81**	− 0.12	0.03	0.06
When I asked my parents about their past, they tell me that there is nothing to really talk about	− 0.14	**0.82**	− 0.21	0.08	0.08
When I asked my parents about their past, they tell me that they don’t want to talk about it	0.05	**0.86**	− 0.10	− 0.02	0.14
Hearing about the suffering my parents went through in their past makes me feel depressed	0.17	0.10	**− 0.64**	− 0.07	0.17
I feel ashamed that I struggle with problems that seem so small or insignificant compared to what my parents went through	− 0.07	− 0.01	**− 0.80**	− 0.03	− 0.06
I feel guilty that I do not carry the same burden as my parents regarding the hardships in their past	− 0.01	0.13	**− 0.82**	− 0.04	0.07
I feel guilty that my parents have sacrificed so much to come to America for our family	− 0.01	0.01	**− 0.86**	0.10	− 0.05
I feel helpless that I cannot help my parents overcome the struggles from their past	0.27	− 0.01	**− 0.53**	− 0.05	0.29
I have felt angry at the injustices that my parents suffered in their home country, refugee camps, or migration journey to America	0.18	− 0.12	**− 0.65**	0.20	− 0.10
I have resentment towards the people (in my home country, refugee camps, in America) who committed violence against my family	0.18	0.05	**− 0.43**	0.07	− 0.08
I often feel insignificant when I compare what I am doing with my life to the sacrifices my parents have made for our family	− 0.09	0.05	**− 0.77**	0.10	− 0.02
My parents compare my life with their hardships in the past and say that I take things for granted	0.16	0.10	− 0.09	**0.81**	− 0.29
My parents have told me that it is important that I learn about how my people (e.g. Chinese, Laotians, etc.) have suffered before coming to America	0.26	− 0.21	0.07	**0.47**	0.29
My parents have told me that my life in America is like a dream compared to the sufferings they went through in their home country or in their migration journey to America	− 0.13	0.03	0.01	**0.97**	− 0.02
My parents have told me to be grateful that I did not have to suffer as they did	− 0.02	− 0.08	− 0.07	**0.79**	− 0.05
My parents often tell me that my worries are nothing compared to the struggles that others in my community have experienced	0.07	0.03	− 0.02	**0.62**	0.26
My parents say that no one will understand the struggles they have been through	0.04	0.12	− 0.12	**0.63**	0.17
My parents tell me to never forget how our family suffered back in my parents’ home country, refugee camps, or migration journey to America	0.08	− 0.16	− 0.04	**0.54**	0.44
My parents have taken me to events or places that display, show the hardships difficulties trauma back in their home country, refugee camps or in the migration journey to America	0.09	− 0.07	− 0.03	0.03	**0.44**
My parents would teach me songs that were about hardships of their home country	− 0.04	0.14	0.10	0.03	**0.71**
My parents would tell me about folk tales or stories from their home country that taught me about the hardships of war, persecution, resettlement, migration, and loss	0.10	− 0.10	0.00	0.12	**0.76**
My parents would tell me stories of their past hardships in light hearted ways such as through bedtime stories, or funny or humorous stories	− 0.14	− 0.04	− 0.09	− 0.01	**0.69**

##### Observing Parental Response to Past Trauma

This scale assessed trauma transmitted through children’s observation of parental responses to past trauma. Children of trauma survivors who participated in the focus groups reported that they frequently observed their parents silently suffering as they dealt with memories or nightmares of the past. This 8-item subscale assesses observations of various parent responses to past trauma, including crying, getting upset, or becoming superstitious, and has an internal consistency of 0.88.

##### Parental Silence on Past Trauma

This 9-item scale assesses parental silence or absence of mentions of their past trauma experiences. Items included parents never mentioning their past trauma, avoiding or redirecting discussions about their past, or denying its relevance. The scale has an internal consistency of 0.87.

##### Internalization of Parent Trauma

This scale focuses on children’s experiences of receiving parental messages of trauma, namely, the internalization of the trauma manifested through various emotions. As a result of receiving messages about parental trauma, children internalized the sense of shame, helplessness, guilt, depression, and anger. This 8-item scale has an internal consistency of 0.90.

##### Sharing as Instruction

This scale assessed the transmission of direct parental messages of past parental trauma. Parents openly shared about their past hardships related to the experiences in their home country, refugee camps, and resettlement in the United States, often with the aim of instruction or guidance. Parents would emphasize the importance of gratitude or acknowledging the sufferings of their family and people while they instructed children about hardships and distress. This 7-item scale has an internal consistency of 0.90.

##### Sharing Through Cultural Channels

This scale assessed parental messages of trauma that were passed down through socialization of culture. Parents shared trauma narratives through various media, including videos, stories, songs, cultural activities, and pictures. Parents would use these channels to share their experiences with their children. This 4-item scale has an internal consistency of 0.73.

### Analytic Plan

To determine the number of factors for the Exploratory Factor Analysis (EFA) we first employed an item removal strategy in which items that cross-loaded on two or more factors with a difference of below 0.10 were removed in sequential order, followed by re-running the process of extraction without the item (Güvendir, & Özkan, 2022). We retained items if the following conditions were met if the factor loadings were 0.40 or greater, had low uniqueness (< 0.5), and had face and content validity. A total of 17 items were removed due to cross loading, with 36 items remaining for the EFA (see Table S1). Items were removed from all 5 factors, which suggests that item redundancy was spread across factors. The factor with the least items removed (1 item) was factor 3, the internalization of stigma. Next, to determine how many factors to retain for the EFA, we conducted parallel analysis that consisted of a Monte Carlo simulation where eigenvalues from the data before rotation were compared with the eigenvalues obtained from a random matrix with the same number of variables and sample size with 100 replications (Velicer et al., 2000). To determine the number of factors to be retained, the point at which the eigenvalue in the simulated data is greater than that in the actual data was identified. Our data indicated five factors that had eigenvalues greater than those generated by the simulated data.

An EFA was conducted using five factors, with principal axis factoring (PAF) and the oblimin rotation, to allow factors to correlate. We used the Kaiser-Meyer-Oklin Measure of Sampling Adequacy (KMO) ≥0.6 and Bartlett’s Test of Sphericity (*p*<.05) to determine the appropriateness of conducting factor analysis. The Kaiser-Meyer-Olkin measures of sampling adequacy was 0.67 and the Bartlett’s test of sphericity was *p*<.001, suggesting the appropriateness of conducting EFA.

### Results

Principal axis factoring (PAF) was initially performed with all 53 items. An iterative process was employed where we first identified items that cross-loaded onto two or more factors and had a loading difference of less than 0.10, which were then removed, resulting in a total of 36 items for the PAF. To determine the factorability of the ITSI, we used the following criteria: (1) the mean of the Kaiser-Meyer-Olkin measure of sampling adequacy which was 0.67 and well above the recommended value of 0.60 (Cerny & Kaiser, 1977), and (2) Bartlett’s test of sphericity, which was significant (χ2 (630) = 1787.17, *p* <.01), which indicated that the items were intercorrelated and suitable for factor analysis. The PAF with direct oblimin rotation revealed 5 components with eigenvalues ≥ 1 (9.27, 4.48, 2.62, 2.32, 1.69), accounting for 56.65% of the variance. A total of 8 items loaded on the first component (*observing parental trauma*), 9 items loaded on the second component (*parental silence*), 8 items on the third component *(internalization of parental trauma*), 7 items on the fourth component (*sharing as instruction*), and 4 items on the fifth component (*sharing through cultural channels*). Table 2 shows the components and corresponding items.

### Discussion

Results indicated that the EFA derived five distinct factors, accounting for over 56.65% of the variance. The five factors, *parental silence*, *sharing as instruction*,* internalization of parental trauma*, *observing parental response to trauma*, and *sharing through cultural channels*, suggest that the pathway of the transmission of parental trauma is multidimensional, and through distinct socialization processes (e.g., direct sharing versus silence, observation, culture). The components identified in the EFA correspond with findings from existing literature on the intergenerational transmission of trauma, which suggests that parents engage in various forms of sharing about past trauma, including open communication and silence (Dalgaard et al., 2016; Esmaeili, 2011). The ITSI thus constructed required additional analyses to examine the factor structure and utility.

## Study 2

The purpose of Study 2 was to confirm the five-factor structure underlying socialization pathways of the transmission of trauma in an independent sample of Lao American young adults. We hypothesized that with more parsimonious constraints imposed by confirmatory factor analyses, the ITSI would still demonstrate a five-factor structure model. We also expected the scales of the ITSI to be positively correlated with young adult depression and somatic symptoms.

## Method

### Study Procedures

Study 2 examined data from a larger project examining mental health attitudes and help-seeking behavior among Asian American young adults. Recruitment occurred in collaboration with community social service agencies in Asian American communities in the Midwest. Partnership with local ethnic community-based social service agencies was developed over several years, building mutual trust through various collaborations. Each organization had deep ties to the communities they served. Thus, partnership with each of these organizations was crucial in recruiting community members.

Participants were informed of the study through the distribution of flyers or verbal announcements in person by research and collaborating agency staff. Agency staff shared the study through informal networks as well as through conducting information sessions on the study with research staff to community members. All participants consented online before completing two online surveys for the original project that took about 60 to 120 min each to complete. Participants were compensated $50 for their time for each survey. All study procedures were approved by the University of Chicago Institutional Review Board (approval number IRB18-0889).

### Participants

Participants included a sample of 223 Lao American young adults (mean age 26.48 years (SD = 6.48), age range 18 to 39, 57.4% female; see Table 3). The median level of education for this sample was high school graduation or completion of a GED, and roughly 71% reported household income of more than $35,000 per year. Approximately 79% of the sample was U.S. born, and the remaining 21% migrated to the United States at the age of 10 or below. All of the participants were fluent in English, and 42% reported fluency in spoken Lao.

**Table 3:** Descriptives for Study 2 Participants

	Sample size (*n* = 223)
Sex, *n* (%)	
Male	95 (42.6%)
Female	128 (57.4%)
Age in years (SD) [Range]	26.48 (6.48) [18–39]
Household Income, n (%)	
Less than 35,000	66 (29.46%)
More than 35,000	157 (70.54%)
Nativity, n (%)	
U.S. born	176 (78.9%)
Foreign born	47 (21.1%)
Education, n (%)	
Less or equal to high school (GED)	52 (23.32%)
Higher than high school	171 (76.68%)

#### Young Adult Mental Health Measures

Depression and Somatic Symptoms. The Depression and Somatic Symptom Scale (Hung et al., 2006), is a 22-item scale that assesses somatic symptoms and symptoms of depression. The Scale has two subscales: the Depression subscale (12 items, internal consistency of 0.91) and the Somatic subscale (10 items, internal consistency of 0.86).

#### Covariates

Covariates, which included demographic variables (i.e., age, gender, household income, education, nativity, and years in the U.S.), were assessed. Gender was dummy-coded with male as a reference. Household income was recategorized into dummy-coded categories, with “less than $15,000 per year” as the reference category. Education was recategorized into dummy-coded categories with “high school graduate or less” serving as a reference. Nativity was coded as 0, foreign-born; 1, U.S.-born, and years in U.S. residency included the number of years the participant has resided in the U.S.

#### Analytic Plan

For Study 2, a series of CFA with 5 factors were conducted to confirm the factor structure derived in Study 1. We specified a correlated five-factor, higher-order factor, and bifactor CFAs based on the results from Study 1. For all CFAs, primary factor loadings were used to achieve a simple structure for the CFAs. Model fit indices were examined to determine the nature of the items assessing the five pathways of trauma transmission. For the correlated five-factor model, each item was specified to load onto one of the five factors, and factors were allowed to be correlated to one another. For the higher-order CFA, items that loaded onto the five lower-order factors, which also shared a common source of variance, were identified as loading onto a higher-order factor. Finally, in the bifactor CFA, all of the items were loaded onto one general factor, and residual variance was explained by the five orthogonal first-order factors.

Model fit was examined by using the chi-squared value for the overall model fit, the comparative fit index (CFI), the Tucker–Lewis index (TLI), the root mean square error of approximation (RMSEA), and the standardized root mean square residual (SRMR). We used the cutoff value of 0.90 and 0.95 for CFI and TLI, for SRMR a cutoff value of 0.08, and for the RMSEA, between 0.05 and 0.10, which is considered a fair to mediocre fit (Hu & Bentler, 1999; Kenny et al., 2015; MacCallum et al., 1996). Reliability, descriptive statistics, and item-total correlations were examined to test the measurement fit of each scale (Nunnally & Bernstein, 1994). Bivariate correlations were conducted among constructs and existing measures of young adult mental health in order to examine construct validity.

## Results

### Confirmatory Factor Analysis

**Fig. 2:**
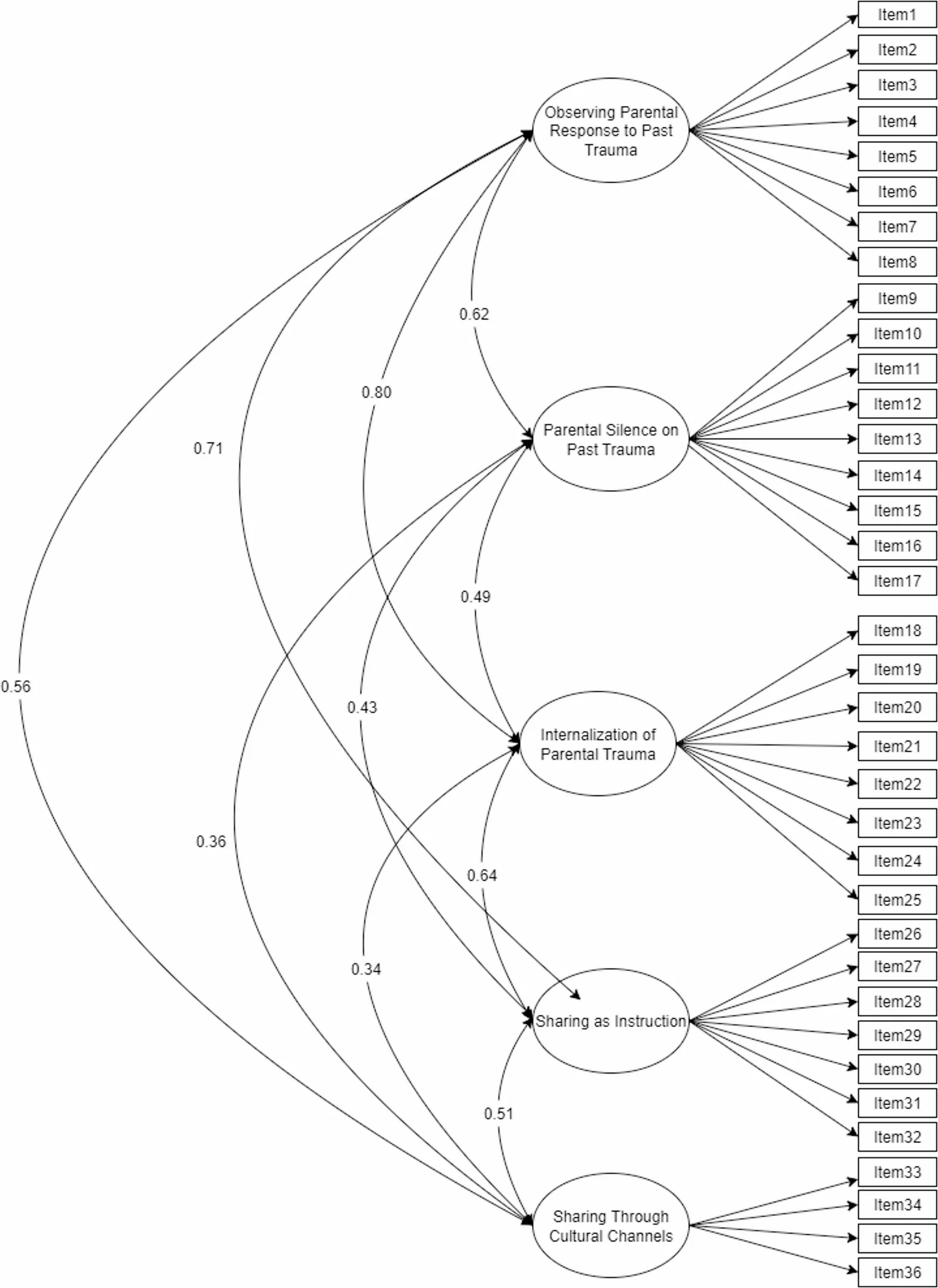
Correlated five-factor confirmatory factor analysis

A series of CFAs were conducted: the five-factor correlated factor structure, a higher order factor structure, and a bifactor structure. For the correlated five-factor CFA, results showed that the model had a good model fit with the data, χ2 (528) = 1038.67, *p*<.05; RMSEA=0.07; CFI=0.90, AIC = 21549.64, BIC = 22008.85. The higher-order factor structure, with components 4 (sharing as instruction) and 5 (sharing through cultural channels) loading on a higher-order construct, indicated a poor model fit (χ^2 (532) = 1068.16, *p* <.05; RMSEA=0.07 CFI=0.89, AIC = 21571.13, BIC = 22016.93). The bifactor model did not achieve convergence. This may be related to a few reasons, first, that there is no one general factor of trauma transmission, and second, the lack of convergence may be related to our smaller sample size as bifactor models tend to perform better in larger sample sizes (e.g., *N* > 800) than smaller samples (e.g., *N* > 200) (Table 4). Overall, the correlated five-factor model indicated the best model fit. This suggests that rather than one general factor that encapsulates parental transmission of trauma, there are multiple socialization pathways through which parental trauma is transmitted. These finding underscores the multidimensionality of parental transmission of trauma (see Fig. 2).

**Table 4:** Summary of the fit indices across three models for the Intergenerational Trauma Socialization Inventory

Model	χ²	df	CFI	RMSEA	AIC	BIC
Five-Factor Correlated Model	1038.67	528	0.90	0.07	21549.64	22008.85
Higher Order Factor Model	1068.16	532	0.89	0.07	21571.13	22016.93
Bifactor Model	Convergence was not achieved					

### Reliability and Correlations

All 36 items were examined for floor or ceiling effects. A review of item descriptives indicated variability in participant responses, with item means ranging from 1.90 to 3.83 and standard deviations from 1.3 to 1.6, suggesting adequate spread of participant responses across the 5-point Likert scale.

For all five scales of parental transmission of trauma, internal consistency ranged from 0.84 to 0.91. Composite scores for each scale were derived by calculating the mean. Pearson’s correlations revealed that the five factors were positively correlated with one another, indicating the interrelatedness of parental pathways of transmission (see Table 5). To determine concurrent validity, the five scales were correlated with young adult mental health outcomes, specifically, somatic symptoms and symptoms of depression. All five factors were positively associated with somatic symptoms and symptoms of depression.

**Table 5:** Correlations between the five factors and young adult mental health outcomes

	(1)	(2)	(3)	(4)	(5)	(6)	(7)
(1) Observing Parental Response to Past Trauma	1.00						
(2) Parental Silence on Past Trauma	0.57*	1.00					
(3) Internalization of Parent Trauma	0.65*	0.43*	1.00				
(4) Sharing as Instruction	0.65*	0.35*	0.55*	1.00			
(5) Sharing Through Cultural Channels	0.50*	0.31*	0.31*	0.51*	1.00		
(6) Depressive Symptoms	0.50*	0.35*	0.39*	0.30*	0.24*	1.00	
(7) Somatic Symptoms	0.46*	0.34*	0.39*	0.26*	0.24*	0.80*	1.00
Mean (SD)	2.85(1.12)	2.37(1.08)	3.21(1.08)	3.34(1.08)	2.29(1.15)	1.95(0.81)	2.21(0.79)
Internal Consistency (Cronbach’s α)	0.91	0.91	0.91	0.89	0.84	0.86	0.86

* shows significance at the 0.05 level

## General Discussion

The purpose of this study was to develop and evaluate the psychometric properties of a new self-report measure of the intergenerational transmission of parental trauma among Lao American young adults. The Intergenerational Trauma Socialization Inventory is one of the first self-report measures that centers on assessing culturally specific socialization pathways of trauma between parents and their children. The five independent yet interrelated factors illustrate that messages of parental trauma are passed down from parents to youth through distinct yet related socialization pathways – such as through parental silence, parental responses to their past trauma, sharing through cultural socialization or parental instruction, and internalization. Our results suggest that the ITSI is a valid and reliable measure of parental socialization of trauma experiences for Lao American young adult children of parents who have experienced significant trauma.

The constructs derived from the ITSI correspond with theorized communication styles of silence and open communication (De Haene et al., 2012). The construct, *parental silence* illustrates the varied ways in which parents engage in silence in response to their past trauma. Parental avoidance of discussing past histories may signal to children their parent’s need to conceal or hide emotionally weighted experiences and, in turn, affect the child’s own emotional state as a form of connection to their parent. Studies on parental suppression or detachment of emotion suggest contagion effects, where parental suppression results in the transmission of stress from parent to child (Ruscio et al., 2002; Waters et al., 2020). Parental responses to silence their trauma may also be influenced by cultural values – in Lao culture, Buddhism has a central influence, and one of its teachings encourages the cultivation of the virtues of perseverance and tolerance to overcome suffering and be reborn in a higher state (i.e., karma) (Hsu et al., 2004). Parental silence in response to trauma could also be a form of the cultural socialization of central cultural values, for example, Nagata and Cheng (2003) reported that Nisei Japanese American parents sought to instill tolerance and perseverance in their children through upholding the Japanese cultural values of gaman, enryo, and shikata ga-nai. Parental silence also includes the absence of mention of past trauma, which may be met with confusion and distress by offspring, and impact psychological adjustment. Correlations between parental silence and children’s somatic and depressive symptoms indicated a positive association, suggesting that parental silence is related to poorer adjustment among Lao American young adult children. Literature examining intergenerational effects of the Holocaust has noted the impact of the “conspiracy of silence,” which has been found to manifest as symptoms of mental health problems among the children of survivors. In a study of Cambodian refugee families, Lin et al. (2009) also found that parental silence affected children’s sense of community belonging and shaped patterns of avoidance passed down to them. Parental silence may be associated with unintended psychological outcomes of offspring, highlighting a central need for effective ways of communicating difficult family histories among trauma survivors.

The construct, *sharing as instruction*, illustrates an explicit form of communication of parental trauma. This form of trauma transmission involves the open sharing of parental histories, providing a parallel to the sharing of trauma through stories or open disclosure (Dalgaard et al., 2016; Esmaeili, 2011). *Sharing as instruction* was the most common form of trauma transmission among Lao families, suggesting that offspring generally grew up hearing directly about their parental trauma. In *sharing as instruction*, parents discussed their past experiences to convey a message of gratitude or to remember familial sacrifice. Sharing of parental trauma in the context of youth’s experiences may reflect culturally specific socialization approaches. For Lao parents, one of the avenues through which they instill Buddhist teachings about cultivating virtuous and moral behavior is through sharing their life stories, including their past trauma. Nagata and Cheng (2003) also found that over 65% of Japanese American parents reported that experiences related to the Japanese internment were a central topic of conversation with their children, with many desiring to educate the next generation about the internment and injustice. Wang et al. (2000) found that Chinese parents’ story narrations or memory conversations focused on encouraging adherence to social values and cultural standards of behavior. Across families who have endured trauma, retelling stories of survival is central to reconnecting with what was lost (Esmaeli, 2011) and fostering pride in one’s culture (Nagata & Cheng, 2003). Correlational analyses for Lao children found that *sharing as instruction* was associated with more somatic and depressive symptoms, which reflects the psychological burden of receiving stories of parental trauma.

Lao American young adults also received messages on their parents’ past histories through indirect pathways, namely *sharing through cultural channels* and *observing parental responses to trauma*. Given that culture is central to how families make meaning of their life experiences, including traumatic ones (Danieli, 2007), cultural channels such as traditional songs, family and historical stories, as well as visiting places of remembrance, are likely a natural medium for families to share about their past. While our sample involved cultural messages of family trauma, these approaches are more broadly illustrative of cultural socialization practices.

*Observing parental response to past trauma* captures how parental reactions to their own trauma triggers can inadvertently be a way in which trauma is transmitted from parents to children. Lao American young adults observed parents reacting to past trauma in various ways, including having nightmares, emotional dysregulation, and silence. These reactions likely reflect parental PTSD responses that children are observing. *Observing parental response to trauma* was significantly associated with young adults’ depression and somatic symptoms. While our findings are cross-sectional, they support existing literature linking parental PTSD with children’s internalizing problems (Field et al., 2013; Shrira et al., 2019).

Finally, *internalization of parental trauma* described Lao American young adults’ response to receiving parental messages on past trauma. This is the only scale that focuses on the child’s experiences of receiving parental messages of trauma. The scale captures Lao American young adults’ feelings of guilt, helplessness, shame, anger, and resentment directly related to hearing about parental trauma. Correlational analyses showed that *internalization of parental trauma* was significantly associated with somatic symptoms as well as depression, suggesting that internalization is associated with the negative adjustment of offspring. Limited research has examined the actual internalization experiences of offspring of trauma survivors, highlighting a need for more empirical investigation.

### Implications for Use

This analysis of the ITSI suggests that this measure can be a useful tool to help both researchers and clinicians understand how culturally specific parental trauma is communicated to offspring, as well as the implications of this communication on adult children’s mental health. Our empirical findings in the EFA, CFA, and correlational analyses support its reliability and its content and construct validity, suggesting that this measure adequately captures how parental trauma is received by offspring. The five scales in the ITSI capture various socialization pathways of parental trauma, ranging from silence to implicit and explicit parental sharing, reflecting its ability to capture the broad range of pathways through which parents may communicate their trauma to their children. While evidence supports the intergenerational transmission of trauma from parents to children, understanding *how* this transmission occurs will be important, particularly for intervention. For example, the ITSI can be included in the initial stages of clinical assessments to identify primary socialization pathways of trauma transmission within the familial context, which can then be areas of target for intervention. Practitioners can use the information gained from the ITSI to helpparents increase their awareness of how they are communicating about past trauma, whether knowingly or unintentionally. For example, a practitioner working with a SEA refugee family may identify how parental silence about trauma, which is construed to align with Buddhist and cultural values of emotional restraint and enduring suffering, may inadvertently affect how offspring perceive closeness to their parents, thereby impacting offspring’s mental health. In such a scenario, the ITSI can help the family identify existing patterns of family communication and processes that can be targeted in intervention, such as enhancing familial cohesion and connectedness. Moreover, by helping parents understand the very processes by which trauma is transmitted, practitioners can intervene in promoting healthy communication pathways that allow for the sharing of familial and cultural histories in ways that are tailored to the child’s understanding. This may be particularly meaningful if applied to community mental health programs that are embedded within the ecosystem of the respective ethnic communities. Moreover, the information derived from the ITSI can contribute to increasing both the recognition and knowledge base of Asian Americans’ histories which continues to be largely ignored in the education of Asian American children and youth. Bringing to light the parental histories and traumas of Asian Americans will be important in helping children and youth develop a culturally and historically grounded understanding of their family and ethnic group’s histories, such as through changes to curriculum in education. In this way, this study presents an initial attempt to capture salient mechanisms of intergenerational transmission of trauma that is likely to be a helpful tool for researchers, clinicians, and policy makers who are serving Asian American families and communities whom historical traumas have been marked, whether in their homeland or during their migration pathways in resettlement.

### Limitations and Future Directions

While the findings support the utility of the ITSI, there are some limitations to consider. The ITSI was developed using data from focus groups with Lao Asian youth and young adults, and thus it will be largely reflective of the trauma transmission experiences of Lao Americans rather than those of Asian Americans more broadly. Moreover, since the original sample was recruited through partnerships with local ethnic community agencies, it will be important to identify whether Lao American families in other geographic locations also share similar experiences of intergenerational transmission of trauma. Future studies should test measurement invariance to determine whether the ITSI can be used with other populations such as with other generations (e.g., first generation immigrant parents), and other Asian American ethnic groups as well as broader immigrant and refugee populations that have experienced historical trauma. Moreover, the pathways of transmission assessed in the ITSI are not comprehensive, and future research is needed to expand on the various socialization pathways related to trauma that occur in families. It is also important to note that the ITSI captures adult children’s reported experiences of receiving messages about parental trauma. Participants’ responses may be influenced by their ability to recall receiving parental messages of trauma. Moreover, since this is a self-report survey, there may be potential bias in the reporting of parental transmission of trauma. It would be important for future research to also assess parents’ reports of the transmission of trauma messages and compare these reports with how their children are receiving these messages. Further, we do not know whether language barriers influenced what and how parents shared about their past trauma histories. It is likely that either the parents’ limited English language fluency or children’s limited understanding of their parents’ native tongue restricts what parents can share and how the young adults received the messages about parental trauma. It will be crucial for future studies to examine the effect of language barriers on trauma transmission. Moreover, the cross-sectional nature of our data also precludes drawing conclusions about the effect of trauma messages on children’s mental health. Our study did not examine the impact of structural barriers that may affect children’s mental health, including acculturative stress (Hwang & Ting, 2008), racial discrimination (Miller et al., 2011), lack of social support or social isolation (McLaughlin et al., 2014). Future studies should examine whether intergenerational transmission of trauma affects the mental health outcomes of the children of trauma survivors over time, above and beyond the impact of structural factors that burden immigrant families. Finally, one of the limitations of our paper is the focus on adverse mental health and psychological outcomes of parental socialization of trauma. In recent years, scholars have noted that multifinality is evident in the aftermath of trauma, with individuals demonstrating resilience and posttraumatic growth, such as higher problem-solving skills, academic functioning, daily life skills, and child prosocial behaviors, and that these may also be intergenerationally transmitted (Howell et al., 2021). As observed in our study noted in above, parents’ socialization approaches are often shaped by a desire to pass onto the next generation, virtues, values, and knowledge, which may foster resilience (Shevell, & Denov, 2021). Thus, future studies should examine whether these parental socialization pathways are related to the fostering of resilience, adaptation, and healing in children whose parents have experienced trauma.

Nevertheless, this study demonstrates that the ITSI is an effective measure of transmission of trauma from parents to children among SEA families with histories of trauma. We have shown the importance of examining the specific socialization pathways of parental trauma and their relation to children’s mental health. Moreover, our measure, derived from focus groups with community members, reflects the need to assess culturally specific pathways of the transmission of trauma. Considering that the healing process that communities experience is culturally anchored, understanding how trauma is transmitted in specific cultural contexts is key to addressing the specific mechanisms of the transmission of trauma across culturally diverse groups and supporting healing in these groups. Such knowledge will be crucial in informing the development of interventions that address the intergenerational transmission of trauma, particularly among culturally diverse communities, both in the United States and globally.

## Declarations

### Public Relevance

The intergenerational transmission of historical trauma from parent to child occurs through multiple pathways of parental socialization, shaping the mental health of subsequent generations.

### Key Findings

Parental socialization is a central mechanism through which parental historical trauma is passed down from parent to child, shaping the mental health of subsequent generations. Through a newly developed measure that assesses parental socialization pathways of trauma among Lao American refugee families, trauma transmission was found to occur through multiple avenues, and each was linked to mental health outcomes of offspring. Our measure can be a useful tool for mental health interventions with refugee populations and, in particular, help understand and intervene with central pathways of trauma transmission that impact the mental health outcomes of the next generation.
